# Single-cell multi-omics sequencing reveals the immunological disturbance underlying Kawasaki disease

**DOI:** 10.3389/fmolb.2026.1758948

**Published:** 2026-01-16

**Authors:** Xue Fan, Shuang Deng, Yuehao Xu, Bin Wang, Xin Guo, Jinwen Liao, Mingguo Xu

**Affiliations:** 1 Longgang District Maternity & Child Healthcare Hospital of Shenzhen City (Longgang Maternity and Child Institute of Shantou University Medical College), Shenzhen, China; 2 Department of Pediatrics, Shenzhen Hospital, Southern Medical University, Shenzhen, China; 3 Department of Pediatrics, The Third People’s Hospital of Longgang, Clinical Institute of Shantou University Medical College (The Third People’s Hospital of Longgang District Shenzhen), Shenzhen, China; 4 Guangdong Key Laboratory for Biomedical Measurements and Ultrasound Imaging, National-Regional Key Technology Engineering Laboratory for Medical Ultrasound, School of Biomedical Engineering, Shenzhen University Medical School, Shenzhen, China; 5 Department of Experiment & Research, South China Hospital, Medical School, Shenzhen University, Shenzhen, China; 6 Department of Pharmacology, Shantou University Medical College, Shantou, China

**Keywords:** immunological disturbance, Kawasaki disease, scATAC-seq, ScRNA-seq, vascular inflammation

## Abstract

**Introduction:**

Kawasaki disease (KD) is an acute autoimmune vasculitis that predominantly affects children under 5 years of age. Although immune dysregulation is considered central to KD pathogenesis, the cellular heterogeneity and regulatory mechanisms underlying this process remain incompletely understood. Single-cell multi-omics technologies provide an opportunity to characterize immune alterations at high resolution.

**Methods:**

Peripheral blood mononuclear cells (PBMCs) were obtained from two children with typical KD and two age-matched healthy controls. Integrated single-cell RNA sequencing (scRNA-seq) and single-cell assay for transposase-accessible chromatin sequencing (scATAC-seq) were performed to characterize immune cell composition, transcriptional profiles, and chromatin accessibility. Comparative analyses were conducted to identify altered immune cell subsets and dysregulated signaling pathways in KD.

**Results:**

Children with KD exhibited marked immune dysregulation, characterized by altered proportions and functional states of multiple PBMC subsets, including T cells, B cells, and natural killer (NK) cells. Notably, specific NK cell subsets were associated with the pathogenesis of intravenous immunoglobulin (IVIG)-resistant KD. Pathway analyses revealed significant dysregulation of toll-like receptor signaling, B cell and T cell receptor signaling, Th17 and Th1/Th2 differentiation, NK cell–mediated cytotoxicity, and platelet activation pathways.

**Discussion:**

By integrating scRNA-seq and scATAC-seq data, this study delineates the heterogeneity of immune cell populations in KD at the single-cell level. The findings highlight coordinated immune and platelet activation pathways that may contribute to KD-associated inflammation and IVIG resistance. These results provide mechanistic insights into KD immunopathogenesis and suggest potential cellular and molecular targets for therapeutic intervention.

## 1 Introduction

Kawasaki disease (KD) is an immune-mediated systemic vasculitis syndrome that primarily affects children under the age of 5 and that can lead to coronary artery lesions (CALs). It has become one of the most common causes of acquired heart disease in children in many developed countries (McCrindle et al., 2017; Noval et al., 2024; Jone et al., 2024). The etiology of KD remains unclear, and diagnosis depends mainly on clinical features (Cannon et al., 2023; Beltran et al., 2023; Wang et al., 2023). High-dose intravenous immunoglobulin (IVIG) administered within 10 days after the onset of a high fever is the standard treatment for KD and can significantly reduce the incidence of CALs. However, approximately 10%–20% of KD patients are resistant to IVIG and have an increased risk of developing CALs (Day-Lewis et al., 2024; Du and Lee, 2023).

The current widely accepted view on the pathogenesis of KD is that it is a clinical syndrome caused by the abnormal activation of the autoimmune system that is triggered by one or more widely existing infectious agents based on certain genetic susceptibility (DeHaan et al., 2024; Noval and Arditi, 2020). The immune response during the acute phase of KD involves the activation of many different components of the innate and adaptive immune systems (Burns, 2024). Researchers have mostly found that dynamic immune disorders occur in patients with KD, including the enhancement of early innate immunity and the imbalance of Th17/Treg/CTL/B cell response in adaptive immunity (Chen et al., 2021).

Single-cell sequencing encompasses genomics, transcriptomics, epigenomics, proteomics, and metabolomics sequencing and differs from bulk sequencing, which provides average data. It is a powerful tool for deciphering cellular and molecular landscapes at a single-cell resolution (Lei et al., 2021; Li and Wang, 2021; Paik et al., 2020).

However, the immunopathological mechanisms of KD—particularly those underlying IVIG resistance—remain insufficiently defined, partly due to the limitations of bulk profiling approaches that obscure cell-type–specific regulatory signals. Given the marked heterogeneity of immune responses in patients with KD, a higher-resolution strategy is required to dissect the transcriptional and epigenetic dynamics at the single-cell level. Single-cell RNA sequencing (scRNA-seq) and assay for transposase-accessible chromatin sequencing (scATAC-seq) enable the simultaneous exploration of gene expression and chromatin accessibility across individual immune cell subsets. Therefore, we performed an integrated scRNA-seq and scATAC-seq analysis to characterize the immune landscape of KD and to uncover molecular features associated with IVIG resistance.

## 2 Materials and methods

Four children aged 3–5 years were enrolled in the study, including two patients with acute KD and two age-matched healthy controls undergoing routine well-child evaluations. Participants were recruited from Shenzhen Children’s Hospital and the Third People’s Hospital of Longgang District. The diagnosis of KD was based on the criteria of the American Heart Association. Two-dimensional echocardiography was performed during both the acute and convalescent phases to assess cardiac function and coronary artery status. Neither KD patient developed coronary aneurysms within 1 month of onset. Clinical characteristics are summarized in Tables 1, 2. Peripheral blood samples (2 mL) were collected from all subjects. KD patients’ blood was drawn during the acute phase and prior to the initiation of intravenous immunoglobulin or corticosteroid therapy.

**TABLE 1:** Clinical information on children with Kawasaki disease (KD group) and healthy children (HC group).

Characteristic	KD group		HC group	
	Patient 1	Patient 2	Case 1	Case 2
Age (years)	4	5	3	3
Gender	Female	Male	Female	Male
Symptoms	Fever, rash, bilateral bulbar conjunctival injection without exudate, strawberry tongue, cervical lymphadenopathy, IVIG resistance	Fever, rash, bilateral bulbar conjunctival injection without exudate, strawberry tongue, cervical lymphadenopathy	Normal	Normal

**TABLE 2:** Laboratory information on children with Kawasaki disease (KD group) and healthy children (HC group).

Laboratory parameter	KD group		HC group	
	Patient 1	Patient 2	Case 1	Case 2
WBC (×10^9^/L)	12.56	12.86	7.9	6.6
NEUT (×10^9^/L)	10.03	9.97	3.9	3.0
LYM (×10^9^/L)	1.62	1.76	3.3	2.6
MONO (×10^9^/L)	0.56	1.09	0.52	0.78

### 2.1 Cell and nuclei suspension preparation and single-cell RNA and ATAC library construction

Blood samples (3 mL per participant) were diluted with an equal volume of phosphate-buffered saline (PBS). Peripheral blood mononuclear cells (PBMCs) were isolated using Ficoll-Paque PLUS at a 1:1 ratio. The cell pellets were resuspended in 1 mL PBS supplemented with 0.04% bovine serum albumin (BSA) and then filtered through a 40-µm Flowmi cell strainer to obtain a single-cell suspension. A hemocytometer was employed to ascertain the cell concentration. The PBMCs were transferred to a 2 mL microcentrifuge tube, and a chilled lysis buffer (10 mM Tris-HCl, 3 mM MgCl_2_, 10 mM NaCl, 0.1% Tween-20, 0.1% Nonidet P40 substitute, 1% BSA) was added to achieve a uniform nuclear suspension.

For the scRNA-seq, cellular suspensions from the patients with KD and the healthy controls were mixed in equal volumes and diluted to a concentration of 1 × 10^6^ cells/mL. Subsequently, the cell suspension was processed on the 10x Genomics platform to generate barcoded complementary DNA libraries for individual cells (Wang et al., 2021a). The scATAC-seq was conducted using the 10x Genomics Single Cell ATAC Reagent v1.1 kit according to the manufacturer’s protocol. Library concentrations were quantified using a Qubit ssDNA Assay Kit. The libraries were sequenced using an MGISEQ-2000 sequencer.

### 2.2 ScRNA-seq data analysis

Raw sequencing data (FASTQ files) were processed using Cell Ranger (v5.0.1) from 10x Genomics to generate a gene expression matrix. Subsequently, downstream analysis was performed using the Seurat R package (v3.2.0). Raw FASTQ files were meticulously filtered, demultiplexed, and aligned to the hg38 reference genome. Subsequently, gene quantification was meticulously carried out using CellRanger (version 5.0.1), yielding filtered gene–barcode matrices. These matrices served as the foundation for the creation of a Seurat object. The scRNA-seq data were subjected to a rigorous standard processing workflow, which included quality control (QC), data normalization, and scaling, all performed using Seurat (version 3.0.2). Cells of inferior quality, characterized by unique feature counts below 200 or exceeding 90% of the most abundant gene counts, as well as those with mitochondrial counts in the top 15%, were meticulously excluded. To identify highly variable genes, we employed the DoubletDetection (version 3.0) package. The default setting returned 2,000 features per dataset. The top 15 principal components were selected for dimensionality reduction and utilized in uniform manifold approximation and projection (UMAP) visualization and clustering analyses. Cell clustering was executed based on the CellMarker database (http://biocc.hrbmu.edu.cn/CellMarker/Index.jsp), leading to the delineation of 17 distinct cell clusters. To uncover differentially expressed genes (DEGs) between these clusters, the FindMarkers function was applied with the default parameters—that is, a Benjamini–Hochberg adjusted p-value of < 0.05 in the Wilcoxon rank-sum test and a log2 fold change greater than 0.25. For each cluster, the marker genes were identified using the FindAllMarkers function in the Seurat package, and then the cell type was annotated by the SCSA method based on published KD-related marker gene sets.

### 2.3 ScATAC-seq data analysis

The scATAC-seq reads were matched to the GRCh38 reference genome and quantified using the CellRanger count pipeline. We used Signac (https://satijalab.org/signac/) to process the QC of cells and peaks with peak_region_fragments greater than 1,000, peak_region_fragments lower than 20,000, pct_reads_in_peaks greater than 15, a blacklist_ratio lower than 0.05, and a nucleosome_signal lower than 10. For each subpopulation-specific peak, the screening criteria were only. pos = TRUE, min. pct = 0.25, and logfc. threshold = 0.25. Nonlinear dimensionality reduction analysis was performed based on the UMAP algorithm, and graph-based algorithms were used to cluster cells into several categories. Each cluster was annotated with marker peaks, the marker peaks were associated with genes, the two most significant marker peaks associated with genes in each cluster were selected for annotation, and the clusters were reannotated with the CellMarker database.

### 2.4 Integrated analysis of scRNA-seq and scATAC-seq datasets

Gene activity was calculated by quantifying fragments within the 2 kb upstream region and gene body, serving as a measure of chromatin accessibility and enabling correlation analysis with gene expression. scATAC-seq and scRNA-seq data were integrated using Seurat’s canonical correlation analysis (CCA) via the FindTransferAnchors function (dims = 1:30, reduction = “cca”), based on the top 2,000 variable genes identified by the FindVariableFeatures function. Cell-type annotations from scRNA-seq were subsequently transferred to scATAC-seq cells using the TransferData function. To generate a co-embedded UMAP, we applied the FindIntegrationAnchors (anchor.features = 2,000, dims = 1:30) and IntegrateData (dims = 1:30) functions in Seurat. Peak-to-gene links were identified following previously described methods (Granja et al., 2019), based on significant trans-correlations after null model adjustment.

### 2.5 Enrichment analysis and peak associated genes

KEGG pathway and GO enrichment analyses of DEGs were completed using the Dr. Tom platform (Shao et al., 2022). By utilizing FindAllMarkers software to analyze the scATAC clustering subgroups, we obtained the marker peaks for each subgroup. The top-ranked peak, defined by the highest avg_logFC value, was selected, and the corresponding genes were assigned according to its genomic position.

## 3 Results

### 3.1 Cell-type annotation of scRNA-seq and scATAC-seq clusters

**FIGURE 1:**
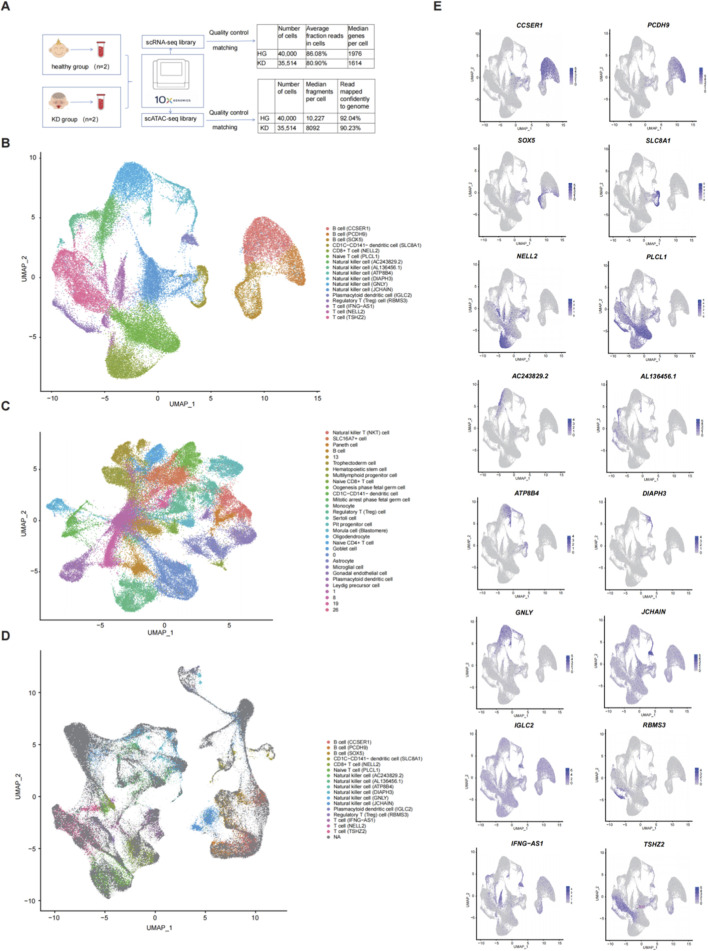
Cell-type identification of single-cell RNA sequencing (scRNA-seq) and single-cell assay for transposase-accessible chromatin sequencing (scATAC-seq) clusters. **(A)** Flowchart of the study and results of the data quality control. **(B)** Uniform manifold approximation and projection (UMAP) plots visualizing clusters of peripheral blood mononuclear cells (PBMCs) derived from scRNA-seq data. **(C)** UMAP plot visualizing clusters of PBMCs derived from scATAC-seq data. **(D)** UMAP plots visualizing clusters. **(E)** UMAP plots showing representative marker gene expression levels in PBMC subpopulations.

We generated scRNA-seq and scATAC-seq profiles for PBMC samples from the healthy control (HC) and KD groups to obtain specific transcriptional profiles for each immune cell subpopulation. After a rigorous QC (Figure 1A), 85,514 cells were clustered into 17 PBMC subgroups in the scRNA-seq data (Figure 1B). The 17 clusters were then annotated into 3 B cells (CCSER1, PCDH9, and SOX5), CD1C–CD141–dendritic cells (SLC8A1), CD8+T cells (NELL2), naive T cells (PLCL1), six natural killer (NK) cells (AC243829.2, AL136456.1, ATP8B4, DIAPH3, GNLY, and JCHAIN), plasmacytoid dendritic cells (IGLC2), regulatory T (treg) cells (RBMS3), and 3 T cells (IFNG−AS1, NELL2, and TSHZ2). We obtained 30 cell clusters generated from the scATAC-seq data, but six subgroups could not be annotated. As shown in Figure 1C, SLC16A7+ cells, monocytes, hematopoietic stem cells, astrocytes, natural killer T (NKT) cells, trophectoderm cells, and oogenesis-phase fetal germ cells were dominant clusters. We found that in the HC group, the SLC16A7+ and trophectoderm cell subpopulations were obviously more abundant than in the KD group, while the situation for monocytes was the opposite (Supplementary Figure S1). In addition, we annotated 17 cell clusters generated from the scRNA-seq data by integrating the scRNA-seq and scATAC-seq matrices. We identified these corresponding scATAC-seq clusters as naive T cells, B cells, T cells, and NK cells, closely resembling the annotation results obtained from the scRNA-seq analysis (Figure 1D). The cell-type–specific marker genes and the proportions of each cell subset are shown in Figure 1E.

### 3.2 Epigenetically and transcriptionally distinct cell subpopulations in KD

**FIGURE 2:**
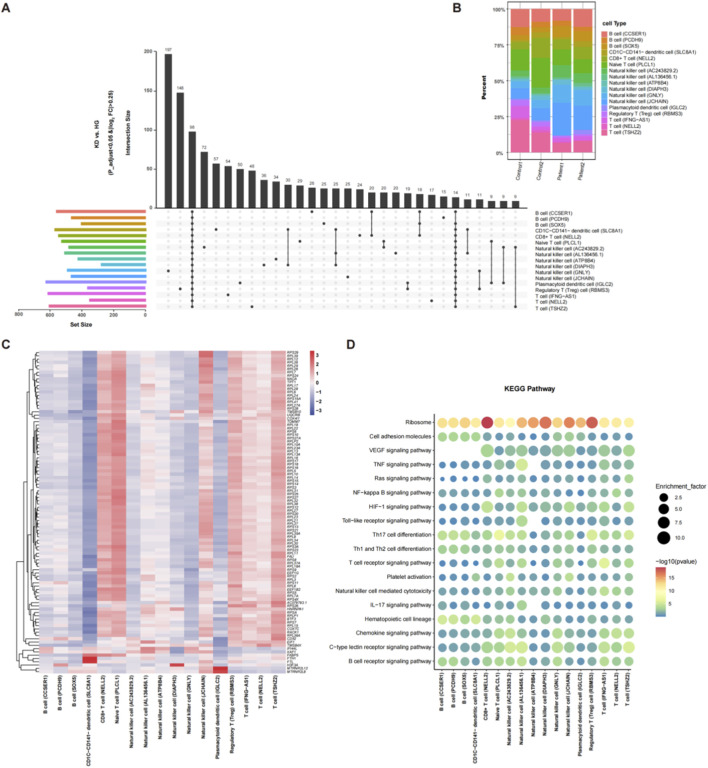
Changes in the biological process of PBMC subpopulations based on scRNA-seq data. **(A)** UpSet diagram illustrating the comprehensive comparative analysis of differentially expressed genes (DEGs) across PBMC subpopulations in the Kawasaki disease (KD) and healthy control (HC) groups. The lateral bars on the left side of the diagram denote the number of DEGs for each PBMC subpopulation. Each hue corresponds to a distinct PBMC cluster. The singular points represent DEGs set to a particular cell group, while the connecting lines signify the overlap of DEGs among different PBMC subpopulations. The vertical bars represent the counts of DEGs within their respective DEG sets. **(B)** Bar chart of the percentage composition of PBMC subpopulations based on scRNA-seq. **(C)** Heat map showing the expression levels of the common DEGs (98 genes) in the 17 PBMC subpopulations. The expression level has been standardized, with red indicating high expression and blue-gray indicating low expression. **(D)** KEGG enrichment analysis of DEGs in all PBMC subpopulations. The dot size denotes the enrichment factor. The color bars show the *p*-values.

To better understand the changes in the biological processes of PBMC subgroups during KD, we assessed the enrichment degree of pathways for each PBMC subset based on the DEG matrix. The findings indicate that among the 18 pathways enriched, 11 were related to the immune system, from the toll-like receptor signaling pathway to the B cell receptor signaling pathway, corresponding to the functional descriptions in the lower central area of the bubble chart. The VEGF signaling pathway was absent for the 3 B cell subtypes. The immune-related pathways for all PBMC subsets included Th17 cell differentiation, the T cell receptor signaling pathway, the chemokine signaling pathway, the B cell receptor signaling pathway, the C-type lectin receptor signaling pathway, Th1 and Th2 cell differentiation, hematopoietic cell lineage, NK-cell−mediated cytotoxicity, and platelet activation (Figures 2A–D).

### 3.3 Comparison of single-cell sequencing results in two children with Kawasaki disease

**FIGURE 3:**
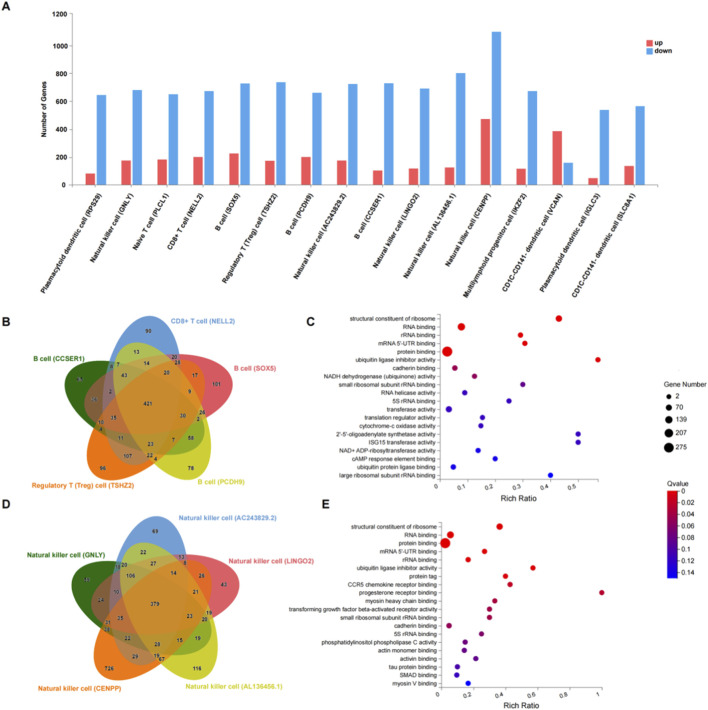
Differences in the molecular function of PBMC subpopulations based on the scRNA-seq data. **(A)** Bar plot showing the DEGs of each cluster in two KD patients. **(B)** Venn diagram of DEGs for 2 T cell clusters and 3 B cell clusters in two patients. **(C)** Bubble chart of GO enrichment analysis for molecular functions (TOP 20) of the intersection of DEGs (421) among 5 T cell and B cell clusters. **(D)** Venn diagram of DEGs for five natural killer (NK) cell clusters in two patients. **(E)** Bubble chart of GO enrichment analysis for molecular functions (TOP 20) of the intersection of DEGs (379) among the five NK cell clusters.

During treatment, two patients with KD were identified: One was IVIG responsive and one was IVIG resistant. To investigate the immunological basis of the treatment response, we compared their single-cell gene expression profiles. Among the 16 identified immune cell subsets (Figure 3A), a total of 3,195 DEGs were detected, with NK cells showing the highest DEG burden (471 upregulated and 1,110 downregulated genes). Subsequently, we performed intersection and union set analyses for five types of B and T cell subsets and five types of NK cell subsets, obtaining 421 and 389 DEGs, respectively (Figures 3B,D). Given the differences in treatment response, we focused on the molecular functions of these DEGs in the GO enrichment analysis. Among the GO terms of the two groups (Figures 3C,E), the majority were related to binding functions. Among the NK cell subsets, there were a significant number of differentially expressed GO terms. Additionally, in the functional enrichment of the two groups, functions such as the structural constituent of ribosome, RNA binding, protein binding, mRNA 5′-UTR binding, ubiquitin ligase inhibitor activity, and cadherin binding were shared by 10 cellular subsets.

## 4 Discussion

KD is a common acute vasculitis in children that usually affects those under 5 years of age. Approximately 25% of untreated children will develop coronary artery aneurysms. KD cases have been reported worldwide, and it is the leading cause of acquired heart disease in children in developed countries (Jone et al., 2024; Rife and Gedalia, 2020; Aggarwal et al., 2023; Watts et al., 2022). Currently, there is increasing evidence that the pathogenesis of KD is caused by abnormal and unbalanced innate and adaptive immune responses (Goel and Yalcindag, 2024; Kumrah et al., 2020; Wang et al., 2021b). Our study aimed to further characterize the cellular and molecular landscapes of acute immune dysregulation in KD through single-cell multi-omics sequencing.

KD exhibits complex immune cell dysregulation, including a range of immune cell types (Giryes and McGonagle, 2023; Saadoun et al., 2021). Using single-cell sequencing technology, it was further confirmed that KD patients have immune cell disorders at the single-cell level (Geng et al., 2021; Sharma et al., 2024; Lin et al., 2024; Liu et al., 2023). We identified different immune cell clusters, including T cells, B cells, NK cells, and dendritic cells. Furthermore, we observed significant differences in the distribution and activation status of these immune cell subsets compared with the HCs. These findings support the idea that KD is characterized by a dysregulated immune response involving multiple immune cell types.

By observing the enrichment degree of pathways for each PBMC subset based on the DEG matrix, we found that most of the enriched pathways were related to the immune system, which is consistent with previous research results. Including the toll-like receptor signaling pathway, the B cell receptor signaling pathway, the T cell receptor signaling pathway, Th17 cell differentiation, Th1 and Th2 cell differentiation, the chemokine signaling pathway, the C-type lectin receptor signaling pathway, hematopoietic cell lineage, natural killer cell-mediated cytotoxicity, and platelet activation, a series of pathways is involved in the immune response process in the acute phase of KD, which may lead to excessive inflammation and vascular damage in this disease (Folga et al., 2023; Chen et al., 2024a; Cao et al., 2023; Schnell et al., 2023).

Although intravenous immunoglobulin combined with aspirin is currently the standard treatment for KD, 10%–20% of children do not respond to this treatment (Broderick et al., 2023; Chen et al., 2023). The early identification of IVIG-resistant KD patients is helpful in providing active intensive treatment as soon as possible, which reduces the rate of IVIG resistance and CAL incidence and improves patient prognosis (Zheng et al., 2024).

Lymphocyte subsets are key components of the innate immune system and important indicators for assessing the function of cellular and humoral immunity (Fan et al., 2021; Kim et al., 2023). Studies have shown that lymphocyte subsets are involved in the development of a variety of immune diseases and can be used to monitor the progression and treatment outcomes of autoimmune diseases (Chen et al., 2024b). We selected two patients with KD: one of whom responded well to IVIG treatment and one of whom did not. Single-cell RNA sequencing analysis of PBMCs from the two KD patients showed that the NK cell subset had the highest number of DEGs and a large number of differentially expressed GO terms, most of which were related to biomolecule-binding functions. Research has indicated that NK cell cytotoxicity and cytokine secretion are related to IVIG-resistant KD (Yang et al., 2024). Studying the mechanisms of NK cells and other lymphocyte subsets in the pathogenesis of IVIG-resistant KD and finding novel marker genes and regulatory networks will provide new directions for the early identification of new therapeutic targets in the future.

Although this approach enabled us to generate hypotheses regarding disease pathogenesis, the present study has several limitations. First, the sample size was limited, and a substantial proportion of low-quality or low-abundance cells was excluded during quality control to ensure analytical robustness. As a result, inter-individual heterogeneity in immune and epigenetic responses may not be fully captured, which may restrict the generalizability of the findings to broader KD populations. Therefore, the current results should be interpreted as exploratory and hypothesis-generating, and further validation through the enrollment of larger, independent cohorts will be necessary to consolidate these conclusions. Second, the analysis relied primarily on omics-based data without orthogonal experimental validation. While high-throughput sequencing provides an unbiased framework for discovery, future studies should incorporate complementary experimental approaches, such as quantitative real-time PCR to validate differentially expressed genes and chromatin immunoprecipitation assays to confirm transcriptional regulatory relationships.

## 5 Conclusion

Integrated single-cell RNA and chromatin accessibility analysis revealed immune cell heterogeneity in children with KD and enabled the investigation of its pathogenesis at a single-cell resolution. Dysregulated pathways related to inflammation and platelet activation were identified. These findings advance our understanding of KD immunopathogenesis and point to potential molecular targets for therapeutic intervention.

## Data Availability

The datasets generated and analyzed during this study are not publicly available due to ethical and privacy restrictions but are available from the corresponding author upon reasonable request and with permission from the Ethics Committee of Shenzhen Children’s Hospital.
