# Supplementary material for: Single-cell multi-omics sequencing reveals the immunological disturbance underlying Kawasaki disease
Source: Front Mol Biosci. 2026 Jan 16;13:1758948. doi: 10.3389/fmolb.2026.1758948 (PMC12855091; doi:10.3389/fmolb.2026.1758948)
Supplement: Supplementary file 1 [file Image1.pdf]

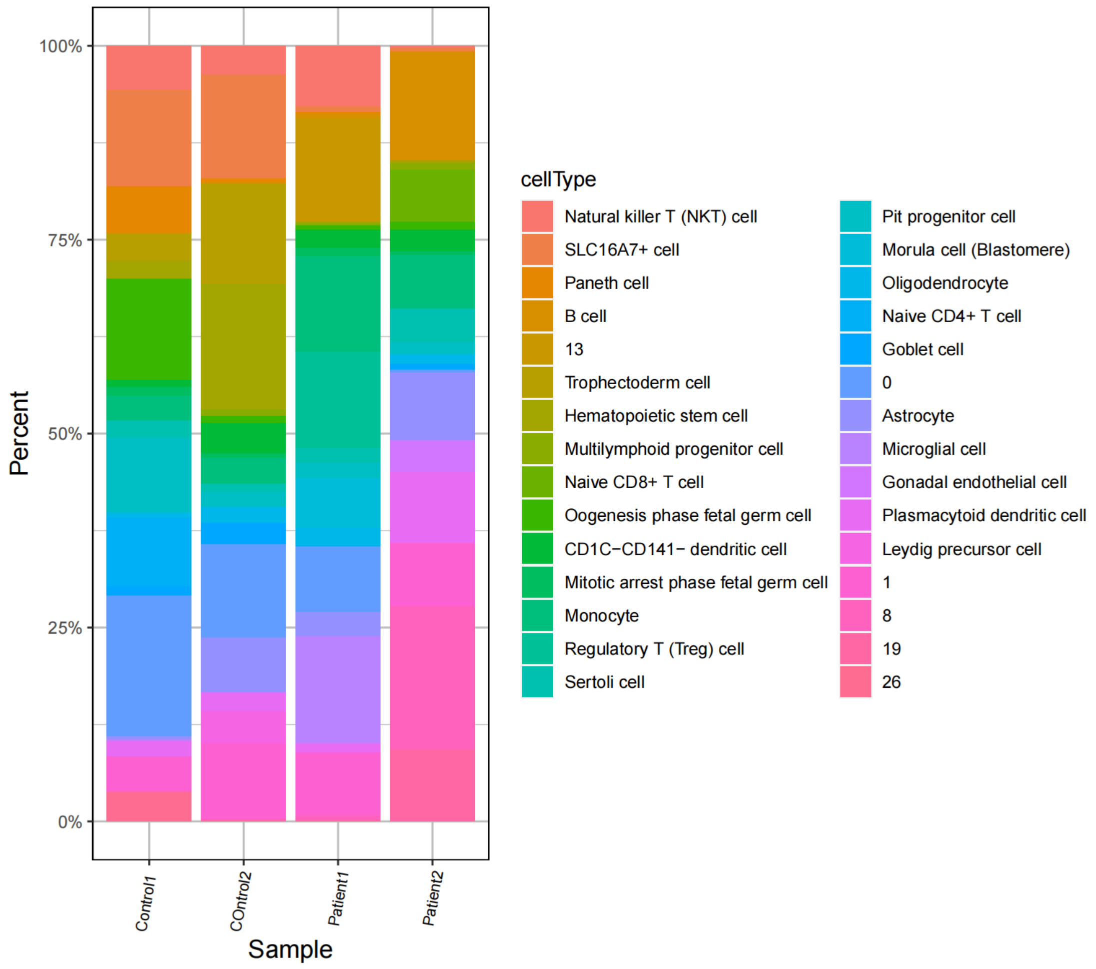

FS1. Relative cell frequencies from the assay for transposase-accessible chromatin sequencing.
